# The Board of Health

**Published:** 1889-05

**Authors:** 


THE BOARD OF HEALTH should establish and maintain as rigid an inspection of the Buffalo street-cars as it would of any other public nuisance that endangered the health of the community.

